# Semi-Solid and Solid Dosage Forms for the Delivery of Phage Therapy to Epithelia

**DOI:** 10.3390/ph11010026

**Published:** 2018-02-26

**Authors:** Teagan L. Brown, Steve Petrovski, Hiu Tat Chan, Michael J. Angove, Joseph Tucci

**Affiliations:** 1 Department of Pharmacy and Applied Science, La Trobe Institute for Molecular Science, La Trobe University Bendigo Campus, PO Box 199, Bendigo 3550, Australia; teagan.brown@latrobe.edu.au (T.L.B.); m.angove@latrobe.edu.au (M.J.A.); 2 Department of Physiology, Anatomy and Microbiology, La Trobe University Bundoora, Bundoora 3083, Australia; steve.petrovski@latrobe.edu.au; 3 Department of Microbiology, Royal Melbourne Hospital, Parkville 3050, Australia; mark.chan3@mh.org.au

**Keywords:** phages, phage therapy, phage formulation

## Abstract

The delivery of phages to epithelial surfaces for therapeutic outcomes is a realistic proposal, and indeed one which is being currently tested in clinical trials. This paper reviews some of the known research on formulation of phages into semi-solid dosage forms such as creams, ointments and pastes, as well as solid dosage forms such as troches (or lozenges and pastilles) and suppositories/pessaries, for delivery to the epithelia. The efficacy and stability of these phage formulations is discussed, with a focus on selection of optimal semi-solid bases for phage delivery. Issues such as the need for standardisation of techniques for formulation as well as for assessment of efficacy are highlighted. These are important when trying to compare results from a range of experiments and across different delivery bases.

## 1. Introduction

An important clinical concern today is that of microbial resistance to commonly used chemotherapeutic agents for infectious disease. Although resistance to antibiotics was documented in the first half of the 20th century, their indiscriminate use and inappropriate application has seen the frequency of resistance escalate [1]. As the number of antibiotic resistant bacteria rapidly grows, the need to find new antimicrobial agents becomes crucial to prevent health care systems from reverting back to the pre-antibiotic era [2]. Antibiotic resistance poses a significant threat to human health, as well as increasing healthcare costs worldwide [2,3]. The issue is recognised as a worldwide crisis needing urgent attention, and, while the instigation of Public Health infectious control and antibiotic stewardship programs have been advantageous, these should be complemented with new approaches and initiatives [1,2,4,5,6]. 

An alternative to antibiotics is the use of lytic phages to eliminate bacterial infection [7]. The first reported use of phage therapy, prior to 1920, was by Felix d’Herelle, the co-discoverer of phage. He applied phages in the treatment of plague and cholera [7,8]. In the early 1920s, the treatment of patients with staphylococcal skin infections, where the phages were injected into and around surgically opened lesions, was published [9,10]. Yet despite these efforts and interest surrounding phage therapy, research in the area was abandoned in Western countries for the newly discovered and convenient antibiotics in the 1940s [11]. Work in phage therapy continued in former Eastern bloc countries and only recently has become more popular worldwide [12]. There are many benefits of using phage in therapy. Their host specificity leads to a targeted lysis of the bacteria involved in the infection and even some biofilms [13]. Further, they are auto “dosing” as their replication leads to an increased titre at the site of infection and they display single hit kinetics. Importantly, phages have low inherent toxicity, and are generally regarded as safe by the US Food and Drug Administration [13]. Additionally, phages will lyse antibiotic resistant strains and are less likely to bring about resistance [13,14]. From 2016, the National Institutes of Health (USA) have funded phage therapy research projects, with the view that these non-traditional therapies could provide strategies to combat anti-microbial resistance [15].

As with pharmaceutical drug delivery, targeting phages to the site of infection remains a hurdle for efficient therapy [12]. There are specific issues that need to be considered when using phages for clinical purposes. For instance, phages are comparatively large biological entities that require structural integrity and viability for efficacy. This requirement poses additional challenges (as compared to delivery of antibiotics/antiseptics which are small chemical molecules) during the formulation process and storage, as well as in the design of protocols for efficacy testing. On the other hand, their ability to replicate results in increased levels at the site of infection, compared to the delivered concentration. This is in direct contrast to antibiotics administrated systemically, whose concentration at the site of infection is significantly less than that administered. Further, while topical antibiotics are usually not recommended because of the risk of resistance development [16], topical phage therapy can be desirable. As such, infection which previously required systemic antibiotic therapy such as skin, soft tissue and surgical site infection can be potentially treated with topical phage therapy. These novel strategies can also be investigated in applications such as topical surgical prophylaxis. 

To date, a diverse range of applications for phage therapy have been reported. Most commonly, liquid preparations are utilised as dosage forms for injections (cutaneous, intravenous, subcutaneous, intrapleural) and local application [14,17]. A wide range of *in vitro* and animal models have shown that delivery of phages for control of bacterial growth is effective in a range of experimental systems. In mouse models, phages have been delivered by diverse routes such as intraperitoneal injection of a three-phage cocktail for treatment of *Klebsiella pneumonia* [18], intraperitoneal injection of *Podoviridae* phages for *Cronobacter turicensis* urinary tract infection [19], and by subcutaneous injection of *Podoviridae* phages in treatment of *Escherichia coli* pneumonia, sepsis and urinary tract infections [20]. In rainbow trout and zebrafish, Columnaris disease was treated with *Myoviridae* phages lytic for *Flavobacterium columnare* in the aqueous habitat [21]. In rabbit models of osteomyelitis caused by *Staphylococccus,* the intraperitoneal injection of a seven-phage cocktail showed efficacy [22] and the intranasal administration of *Podoviridae* viruses lytic for *Pseudomonas aeruginosa* was effective in ameliorating haemorrhagic pneumonia in Mink [23]. Yet more basic research as well as the accumulation of data from extensive clinical trials is required in order to provide comprehensive evidence which will allow approval of phage therapy by regulatory bodies such as the US Food and Drug Administration and the European Medicines Agency [24,25]. This paper reviews some of the known data regarding the formulation of phages in semi-solid and solid dosage forms for the targeting of bacteria associated with epithelial surfaces. It discusses specific issues relating to these formulations and highlights the need for some standardisation in assessment of efficacy of these delivery mechanisms.

## 2. Formulations of Semi-Solid and Solid Dosage Forms for Delivery to Epithelia

Semi-solid emulsions such as creams and ointments are very useful in the delivery of medicaments to epithelial surfaces such as the skin. They tend to be minimally irritating on the skin, easy to apply, easily removed with soap and water, stable enough to avoid the need for frequent applications, and bacteriostatic [26]. Pastes are semi-solid carriers which are often “fatty” and quite stiff in consistency [27]. Their use is often in the treatment of oozing lesions, where their heavy consistency confers a degree of physical protection, and absorptive properties allow them to absorb secretions from wounds. From a patients’ perspective, the more hydrophilic bases may be preferred as vehicles for therapy applied to the facial region, as they are easier to apply and less greasy than the more hydrophobic ointments. The thicker pastes are indicated where there is the need for application to moist surfaces such as oozing wounds, or inside the oral cavity, where other vehicles would be easily removed [27].

Suppositories are solid dosage forms, tapered at one end, which can carry medicaments into epithelial cavities such as the rectum, vagina or urethra. After insertion, these formulations become soft and disperse. Troches (also known as lozenges or pastilles) are solid dosage forms for the delivery of medication orally. They can deliver medicaments to epithelial surfaces such as the oral cavity, oesophagus and gut. They are placed in the mouth, and at temperatures approaching 37 °C they slowly dissolve, liberating the active ingredient [27]. 

As with the formulation of any pharmaceutical drug into a semi-solid dosage form, it is important that phages are incorporated into the vehicle such that there is homogenous distribution throughout the final product. This allows mixture uniformity and ensures consistent delivery of the medicament. In industry this process is undertaken using large scale mixing equipment [27,28], but for the preparation of phage formulations in semi-solid and solid dosage forms as described here for research purposes, the reliance is on small scale mixing equipment and strategies. If results of research into delivery of phage therapy by semi-solid and solid dosage forms are to be reliable, then the outcomes of the small scale mixing processes should be consistent with those of large scale mixing, that is, mixture uniformity, to ensure consistent delivery of the phages. An important issue, however, is that phage structures (e.g., phage tails) may be compromised during large scale mixing processes. Therefore, large scale manufacturing processes have to be verified by industry to demonstrate phage efficacy equivalent to small scale "in-house" mixing processes in research settings.

Geometric dilution is a commonly used technique when low-dose active pharmaceutical ingredients (API) are blended into formulations. It implies the gradual addition of equal portions of the diluent to the API upon blending [29]. The process is an effective way to enhance the equal distribution of the API within the blend, and an increase in mixing time promotes better distribution of the active ingredient [30]. In the formulation of small volumes of concentrated phage solution into semi-solid vehicles, as reported by Brown et al. [31], the process involves the addition, mixing (for at least five minutes) and even distribution of the phage solution into a small portion (approximately 1–2 g) of the semi-solid vehicle, then thorough mixing (for at least five minutes) of this small portion with an equal mass of fresh vehicle. This process is repeated until all the fresh cream has been incorporated, and ensures that the medicament (in this case, the phage) is evenly dispersed throughout the cream. The mixing of phages into solid dosage forms such as troches and suppositories present specific issues, which will be discussed below. 

## 3. Stability of Phages in Semi-Solid Preparations

In the west reports of testing of a phage in a semi-solid preparation suggested that *Myoviridae* phages lytic for *Staphylococcus aureus* incorporated into a commercial cream (at a concentration of 10^8^ PFU per gram) were capable of clearing bacterial cells in broth following insertion of a strip of the cream and a four-hour incubation period at 37 °C [32]. Adequate controls for these experiments were not shown, and it was unclear whether preservatives within the bismuth-based commercial cream had any effect on bacterial growth. In a subsequent study, a commercial cream containing paraffin mineral oil was diluted with water to obtain a lotion, into which were mixed *Podoviridae* phages lytic for *Acinetobacter baumannii* (at a concentration of 10^8^ PFU per gram). In these experiments there was no discussion on the possible effects of preservatives in the commercial cream on bacterial growth. The results showed that while such a lotion was capable of killing bacteria, the lytic capacity was not maintained after 30 days storage of the phage lotion at room temperature [33]. More recent studies had combined commercial burn wound care products with *Myoviridae* and *Podoviridae* phages which were lytic for *A. baumannii*, *P. aeruginosa* and *S. aureus*, then assessed the effect on phage stability and viability following 24 h at 37 °C. These commercial products included creams, gels, suspensions and ointments, and assays involved the dilution of these products 1:1 with phage suspensions. Therefore, the study did not investigate the capacity of intact semi-solid preparations to deliver viable phages, but did demonstrate that some of the active ingredients in the commercial products, such as antibacterial agents, and in particular, acidic compounds, had an adverse effect on phage viability [34]. While this study provided important insight into possible issues when formulating phages into commercial preparations for bacterial control, it highlighted the potential difficulty in dissociating phage effects from other antibacterial effects when such formulations are used. Also, the issue of overuse of antiseptics and antibiotics and potential spread of antimicrobial resistance in bacteria is not addressed with such formulations. To this end, the current phagoburn Phase I/II clinical trials (NCT02116010) which aim to treat burns victims with wound infections caused by *E. coli* and *P. aeruginosa* are employing phage cocktails as the sole antimicrobial agents [35,36].

It is important to ascertain the stability of phage preparations in semi-solid formulations in order to deliver efficacious phage therapy in such carriers. Testing of these formulations should incorporate assays to determine thermo-stability and photo degradation over time [27,28,29]. The most extensive stability tests of such phage formulations that have been reported to date have included storage of the formulations at 4 °C, 20–25 °C and 45 °C in light-protected bottles to ascertain the thermostability and exposure to 50 Lux of light, the standard illumination of a typical room, at 20–25 °C to test the phages photodegradation over time [37]. The quantitation of phage lytic activity following such assays is important. A relatively straightforward quantitative method for this is to measure a standard small weight of the phage cream preparation, and then dilute this into an aqueous solvent such that there is even dispersal of the phage particles throughout. The numbers of active phage particles can then be determined by serial dilution and counting of plaques on a bacterial lawn [37]. One of the issues, however, is that such a method relies on the miscibility of all the components of the semi-solid preparations in the solvent, and so may be more suitable for phage formulations in more hydrophilic creams [31]. The use of a benign aqueous solvent (e.g., water, physiological saline or phosphate buffered saline (PBS)) is important here, as other solvents may prove toxic to bacteria growing on agar plates or the phages contained in the formulation. In more lipophilic ointments, such even dispersal of the components in the aqueous solvent is difficult, and so any count of phage plaques may be skewed, and therefore not a true representation of particle numbers (see below). An alternative method for quantitation may involve measurement of zones of clearing surrounding phage cream strips placed upon a bacterial lawn, as employed by agar diffusion tests in the determination of antimicrobial efficacy. 

Agar diffusion tests such as the Kirby-Bauer disc diffusion method are well established and adopted by International bodies such as the Clinical Laboratory Standards Institute (CLSI) and the European Committee on Antimicrobial Susceptibility Testing (EUCAST) [38]. Such assays require strict standardisation of parameters including diffusion of antimicrobial agent, interaction between media and antimicrobial agent, hydration of media etc. and bodies such as CLSI are instrumental in establishing guidelines to guarantee quality control in such methods [39,40]. The rigorous scientific assessment of phage stability and efficacy in semi-solid formulations is as yet in its infancy, and not subject to such stringent guidelines. Yet standardisation of phage stability and activity in semi-solid preparations is an important issue, and one which requires more research effort to ensure the efficacy of future phage therapy applications using such carriers. It is important to note, however, that the antibiotic testing methodologies mentioned above are for testing purified antibiotic molecules only, and not antibiotics in formulations. Regulatory bodies may adopt current guidance such as potency testing for cellular therapy products and biologics to guide development of standards for bacteriophage formulation efficacy testing [41].

**Figure 1:**
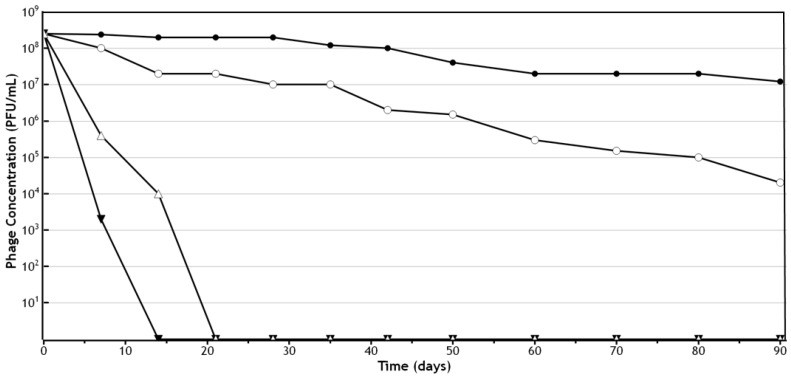
Quantitative assessment of *P. acnes* phage (PAC1) lytic activity and stability in cetomacrogol cream aqueous following storage at various temperatures and light exposures [31,43]. Log scale shown, with data points representing the mean of three samples. ● indicates storage at 4 °C in a light protected bottle; **○** indicates storage at 20–25 °C in a light protected bottle; Δ indicates storage in constant light at 20–25 °C; and ▼ indicates storage at 45 °C in a light protected bottle.

**Figure 2:**
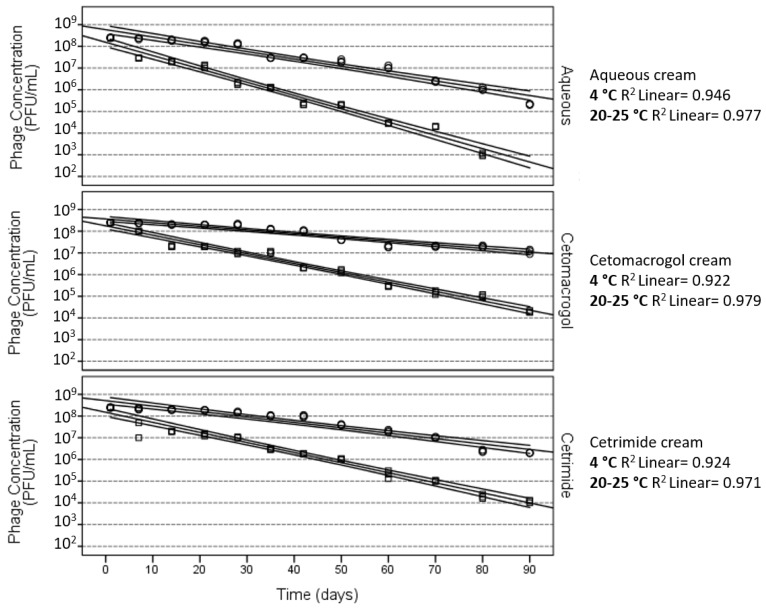
*P. acnes* phage PAC1 stability in semi-solid dosage forms. These regression plots show 4 °C (as depicted by the circles) and 20–25 °C (as depicted by the squares) treatments for each cream type. The central lines in each plot are the regression best fit lines and the curved lines define 99% confidence bounds. For *P. acnes* phage PAC1 cetomacrogol was the optimal semi-solid preparation tested [31].

**Figure 3:**
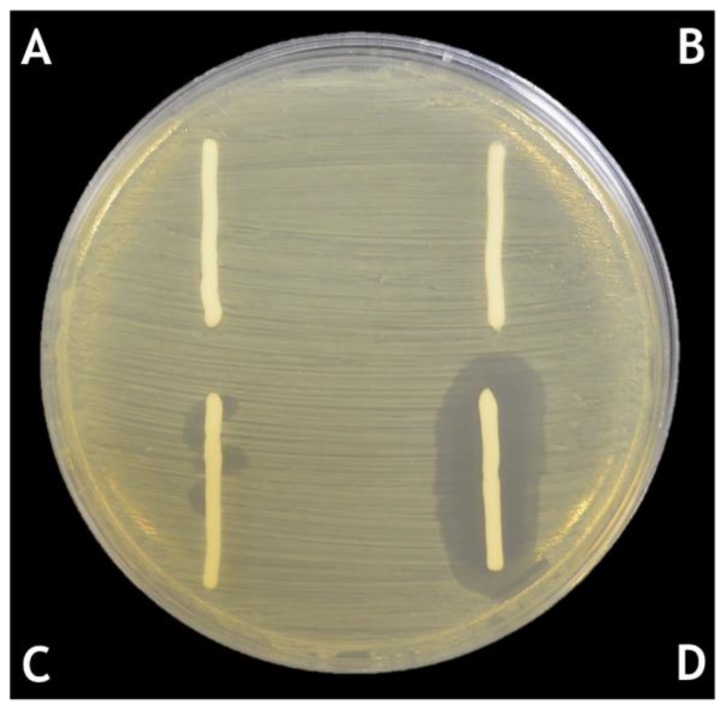
Lytic capacity of the *P. acnes* phage PAC1 cream formulation. *P. acnes* phage formulated in cetomacrogol cream aqueous on a lawn of *P. acnes* bacteria [37]: (**A**) cetomacrogol cream aqueous; (**B**) cetomacrogol cream aqueous with PBS; (**C**) phage cream, PAC1 at a concentration of 5.0 × 10^3^ PFU per gram of cream; and (**D**) phage cream, PAC1 at a concentration of 2.5 × 10^8^ PFU per gram of cream.

The quantitative assessment of phage stability and lytic activity over time (determined by counting of plaques following mixing of small amounts of phage cream 1:100 into PBS, as described above), were employed for *Propionibacterium acnes Siphoviridae* phages formulated into more hydrophilic cream bases (cetomacrogol cream aqueous, aqueous cream, and cetrimide cream aqueous) [31]. These assessments showed that storing the formulations at 4 °C in light-protected containers resulted in the greatest levels of viable *P*. *acnes* phages after 90 days, and that the next best conditions were storage at room temperature (20–25 °C) in a light-protected container [37,42]. Storage at 45 °C and at constant light at 25 °C resulted in a rapid decline in efficacy for the *P. acnes* phage creams (Figure 1). Cetomacrogol cream aqueous provided optimal retrieval of lytic *P. acnes* phage numbers in these quantitation assays (Figure 2 and Table 1). It is important to note, however, that these stability tests assess *Siphoviridae* phages specific for *P. acnes,* and other phages may behave differently in these formulations. These quantitation assays also do not necessarily reflect the capacity for the cream to adequately deliver phages for therapy. For instance, after 90-day storage, cetrimide cream scored more highly than aqueous cream in quantitation assays, yet after this time, although cetrimide cream was a repository of viable *P. acnes* phage particles, these were shown to not be released and lyse underlying and surrounding bacteria when the cream was placed onto a *P. acnes* bacterial lawn on agar (Brown, unpublished data; see discussion in Section 4 for elaboration). In comparison, *P. acnes* phages in cetomacrogol cream aqueous were released and lysed bacterial cells when a strip of the phage cream was placed upon a *P. acnes* bacterial lawn on an agar plate (Figure 3), and this clearing was seen even following 90-day storage at 4 °C. These authors also showed that when *P. acnes* phages were triturated into formulations of cetomacrogol cream aqueous that had been prepared with and without preservative (0.1% chlorocresol), there was minimal effect of the preservative on bacterial survival, and on phage lytic activity and efficacy (Brown, unpublished data).

## 4. Effect of the Ionic Nature of the Semi-Solid Base

Determination of which semi-solid formulation is most suitable for the incorporation of phages is important. Early studies suggested that phages are negatively charged from pH 3.6 to 7.6 [44]. Agarose gel electrophoresis of T7 *Podoviridae* phages demonstrated that the capsids held a negative charge whereas the tail fibres were positively charged according to their migration [45,46]. The immobilisation of phages onto silica particles through electrostatic interactions has shown that *Myoviridae* and *Siphoviridae* phages targeting *Escherichia coli, Salmonella enterica, Listeria monocytogenes* and *Shigella boydii* adsorb to positively charged silica surfaces [47]. The authors hypothesised that the negatively charged capsids bound to the silica surface, “head down” such that the tails were able to bind the bacteria. The biocontrol of *Listeria* and *Escherichia* via immobilisation of phages onto cationic cellulose membranes [48] also suggested the capacity for binding to cations by the negative capsid. The results indicated that the phages capsids were attached to the membrane and the tail fibres were available to target the bacteria. Based on these findings, we surmised that the *Siphoviridae* [31,37] and *Myoviridae* (Brown, unpublished data) phages used in our experiments would contain charged capsids and tails, and as such there may be interactions between the phages and cream formulations. Ionic polymers within some cream bases have the potential to interact with phages through electrostatic forces. Therefore, the ionic nature of the cream may be important in terms of allowing “release” of the phages to access the bacteria in underlying tissue upon which it is spread. In the testing of cetomacrogol cream aqueous (a non-ionic base), aqueous cream (an anionic base) and cetrimide cream aqueous (a cationic base) as carriers of phages, quantitative analysis of viable *P. acnes* phage numbers following storage for 90 days showed that viable phage numbers in cetomacrogol cream were significantly higher than in either aqueous or cetrimide creams (*p*-values < 0.001). Under the same conditions, viable *P. acnes* phage numbers in cetrimide cream were significantly higher than in aqueous cream (*p* < 0.001) [31]. It is important to note, however, that such quantitative analysis involves dispersion and 1:100 dilution of the phage cream in aqueous buffer, then assessment of viable phage by plaque numbers. Dispersion of the cream matrix in an aqueous solution is likely to change the release profile of the phage, as the interaction of dispersed polymeric ions from the cream and phage will be weaker than the interactions of phage within the polymer network of a non-dispersed cream. As such, quantitative analysis may reflect the “storage” of the phages in the cream matrix, and not necessarily the capacity of phage to be released from the cream and to lyse underlying bacteria. Evidence of this was seen following formulation of *P. acnes* phages into the cationic cetrimide cream, where while there may be clearing of the bacterial lawn initially after formulation, this clearing was not seen when a sample of the cream was placed upon the bacterial lawn a week or so later (Brown, unpublished data), even though quantitative analysis showed that there were still viable *P. acnes* phages present in the cream. Therefore, it would seem prudent to assess phage stability in creams by quantitative measures of their viability following storage over time, as well as qualitative functional assessment of the phage cream’s capacity to kill underlying bacteria *in vitro*, as the latter may be a more realistic depiction of the capacity of the phages to be released from such cream formulations. 

## 5. Lipophilicity of the Semi-Solid Base to Be Used for Phage Formulations

Hydrophobic ointments may potentially be useful in delivery of phage therapy to wounds and ulcers, where their capacity to adhere to the wound and occlusive nature may be more appropriate than formulations which are more hydrophilic. Quantitative assessment of the capacity for lipophilic ointments to stably maintain viable phage numbers over time is not as straightforward as for creams. This is because, unlike hydrophilic creams, the ointments do not solubilise and disperse evenly in an aqueous solution, thus making the determination of plaque forming particles per unit volume difficult [31]. Thus, experiments have focussed on qualitative assessment of phage lytic activity following storage over time [31]. Whereas *P. acnes* phage creams demonstrated lytic capacity for at least 90 days, the more hydrophobic emulsifying ointment and simple ointment white were capable of supporting *P. acnes* phage lytic activity for shorter periods of time. For the simple ointment white, this was 50 days following storage at 4 °C in light-protected containers, while storage at 45 °C or constant light exposure yielded no activity by 14 days. For the *P. acnes* phages formulated in emulsifying ointment, phage efficacy was lost by seven days, even after storage at 4 °C [31]. 

It is possible to speculate why these hydrophobic bases (emulsifying ointment in particular) do not apparently support *P. acnes* phage lytic activity as do the more hydrophilic bases. It is possible that the hydrophobic components in the emulsifying ointment may not partition well with aqueous phases such as the aqueous PBS which is the carrier for the phages in these experiments. Another issue is that the emulsifying ointment contains sodium lauryl sulphate, a surfactant capable of denaturing protein and which has been shown to act as a virucide [49]. In this formulation, it may have contributed to disruption of the phage protein coat. While sodium lauryl sulphate is also a component in the formulation of aqueous cream, the concentration in emulsifying ointment is more than three times higher [50]. This could explain why the phage viability is not as dramatically affected in aqueous cream compared to emulsifying ointment (aqueous cream has previously been mentioned as capable of supporting phage viability for at least 90 days) [31]. Another issue is that the thickness of the ointments may have an inhibitory effect on phage release, as compared to the creams. In support of this possibility, it has been noted that the composition of an agar plate surface can affect the morphology of phage plaques, suggesting that a reduced capacity to form extensive clearing zones may reflect physical barriers within the media [51].

Semi-solid formulations which are thicker than ointments, such as pastes, may not be optimal bases for the delivery of phage for therapy. For instance, *P. acnes* phages formulated into zinc paste did not show capacity to lyse surrounding bacteria in an *in vitro* model [31]. To investigate this further, *P. acnes* phages were formulated alone into white soft paraffin (a component of zinc paste), as well as white soft paraffin with the other components of zinc paste: starch and zinc oxide. Lysis of the *P. acnes* bacteria was observed with the white soft paraffin alone and white soft paraffin containing starch formulations [31]. White soft paraffin is a relatively unreactive substance [29], and despite its hydrophobicity, did not adversely affect *P. acnes* phage release and lytic capacity. Starch appeared to mildly inhibit release of the phage, possibly because of the thickness it imparts (as described above), and the potential for starch macromolecules to entangle phage structures such as tails. The zinc oxide was inhibitive of *P. acnes* phage release, possibly because of the sorption of phages to the surface of zinc oxide particles. Metal oxide particles are known to form strong surface complexes with large organic and charged macro-molecules, proteins and even bacteria [52]. 

## 6. Solid Formulations for the Delivery of Phages 

While there are reports of availability of commercial solid dosage forms for delivery of phages to infections of the epithelia [14,17,53], there is scant scientific literature on testing for efficacy and phage stability of such products. Dosage forms such as troches offer potentially attractive approaches to treating bacterial infections of the oral cavity, oesophagus, stomach and subsequent intestinal tracts. Preparation of phages in aqueous solutions are potentially useful in the treatment of oral infections [12,17,54,55]. However, delivery of phages over a sustained period would be optimal, and troches as a vehicle offer such a delivery profile. Indeed, research into treatment of oral infections suggests that application of medicaments in delivery forms such as troches is preferable to delivery via suspensions or solutions [56], which are subject to inherent variability in exposure of the drug to the oral cavity prior to patients swallowing or releasing the fluid from the mouth. While solid substances at 4 °C, troches begin to melt at temperatures approaching 37 °C, and testing for the efficacy and stability of phage troches has shown that at 37 °C, the temperature which is found in the oral cavity, these formulations are able to release lytic *Klebsiella oxytoca* (*Myoviridae*) and *Rhodococcus equi (Siphoviridae)* phages in an in-vitro model, and that the phage lytic capacity was maintained for at least 49 days when troches were stored at 4 °C [31,42]. 

It is important in these formulations that the phage particles are homogenously distributed throughout the final product. This requires particular attention, as, unlike semi-solid preparations which can be triturated during and after addition of phages at room temperature, the troche formulation is heated to approximately 60 °C (a temperature that would inactivate most phages) in order to allow miscibility of the constituents, and then solidifies upon cooling to temperatures of approximately 25 °C (Professional Compounding Chemists of Australia). Therefore, while still molten, but following cooling to temperatures which will minimise damage to the phage particles, the phages and the formulation need to be mixed until homogenous, then poured into moulds and allowed to set. 

The ingestion of troches with lytic phages as the medicament could prove to be a useful way to treat bacterial infections within the stomach, or to modulate intestinal flora. Research has shown that *K. oxytoca Myoviridae* phages are capable of surviving in environments simulating the gastric chamber. Specifically, following 30 min exposure of 10^8^ PFU/mL of *K. oxytoca* phages to simulated gastric fluid at pH 2.5, in which time the phages may be expected to have passed through the stomach if ingested via a troche, there were still significant numbers (10^5^–10^6^ PFU/mL) of viable *K. oxytoca* phages detected [42]. As low gastric pH significantly reduces phage numbers, the co-administration of a proton pump inhibitor (PPI) drug, such as rabeprazole, which may have lower incidence of Phase I/II drug interactions than other PPI, which have been used to assist in phage gastric survival [57], may enhance phage viability. Such medications are very commonly used for gastric reflux, and have the capacity to decrease stomach acidity to pH 6 [58]. Yet while in some patients PPI may increase the risk of infections such as gastroenteritis [59] and pneumonia [60], this approach may alleviate the need for microencapsulation of phages, which is being considered as a method of formulating phages for improved viability in the gastric chamber, as well as to retain a high infective dose in the targeting of a range of tissues downstream [61,62]. Once viable phages have passed through the stomach, they will encounter bile salts in the duodenum, but as has been shown, they are not likely to be adversely affected by such emulsifying agents [42,63,64].

For the delivery of phage therapy to infections in the lower intestinal and genito-urinary tracts, suppositories/pessaries are useful vehicles [27]. As with the preparation of troches, formulation of suppositories requires heating to high temperatures (100 °C) to allow dissolution of constituents, then cooling for pouring into moulds. Therefore, phages need to be added and thoroughly triturated prior to solidification, but following cooling to temperatures which will not destroy the viral particles. *K. oxytoca (Myoviridae)* and *R. equi* (*Siphoviridae*) phages formulated into these delivery forms have been shown to be stable and viable in in-vitro models for at least 56 days when stored at 4 °C [31,42]. 

In conclusion, a range of formulations are capable of successfully delivering lytic phage particles for bacterial killing *in vitro*. These include semi-solid preparations such as creams and ointments, as well as solid preparations such a troches and suppositories/pessaries. Phages are most stable when stored at 4 °C in these preparations, and remain active for at least 90 days in some formulations. Factors which may influence the efficacy of phages in semi-solid preparations include the ionic nature of the cream, as well as the thickness and stiffness of the formulations. Finally, the standardisation of methodologies for assessment of efficacy of phages in these formulations is required.

## Figures and Tables

**Table 1:** *P. acnes* phage PAC1 formulated into semi-solid creams. Mean differences in phage concentration (log_10_) in the various cream types after storage for 90 days at 4 °C. For *P. acnes* phage PAC1 cetomacrogol was the optimal semi-solid preparation tested [31].

Cream (I)	Cream (J)	Mean Difference (I–J)	Standard Error	*p*-Value
Aqueous	Cetomacrogol	−1.329	0.080	<0.001
Aqueous	Cetrimide	−0.868	0.080	<0.001
Cetomacrogol	Cetrimide	0.461	0.078	<0.001
